# Spontaneous uterine rupture with amniotic sac protrusion during the third trimester of a unicornuate uterus pregnancy: A rate case report

**DOI:** 10.1097/MD.0000000000037445

**Published:** 2024-03-15

**Authors:** Yin Yin, Linlin Wang, Zhihong Shi, Yuxin Ma, Juan Yina

**Affiliations:** a Department of Ultrasound, Jinan Maternity and Child Care Hospital Affiliated to Shandong First Medical University, Jinan, China; b Medical Imaging Department, Central Laboratory, Jinan Key Laboratory of Oral Tissue Regeneration, Jinan Stomatological Hospital, Jinan, China.

**Keywords:** ultrasonic diagnosis, uterine rupture

## Abstract

**Rationale:**

Uterine rupture is an obstetrical emergency associated with severe maternal and fetal mortality. It is rare in the unscarred uterus of a primipara.

**Patient concerns:**

A 25-year-old woman in her 38th week of gestation presented with slight abdominal pain of sudden onset 10 hours before. An emergency cesarean section was done. After surgery, the patient and the infant survived.

**Diagnoses:**

With slight abdominal pain of clinical signs, ultrasound examination showed that the amniotic sac was found in the peritoneal cavity with a rupture of the uterine fundus.

**Interventions:**

Uterine repair and right salpingectomy.

**Outcomes:**

After surgery, the patient and the infant survived. The newborn weighed 2600 g and had an Apgar score of 10 points per minute. Forty-two days after delivery, the uterus recovered well.

**Lessons:**

Spontaneous uterine rupture should be considered in patients even without acute pain, regardless of gestational age, and pregnancy with abdominal cystic mass should consider the possibility of uterine rupture.

## 1. Introduction

Uterine rupture refers to the rupture of the body, bottom or lower part of the uterus during delivery or late pregnancy, which directly threatens the life of parturient and fetus, and is a critical obstetric disease. It is rare in the unscarred uterus of a primipara. We present a case of a spontaneous complete uterine rupture at a gestational age of 38 weeks in a 25-year-old patient as follows.

## 2. Case presentation

**Figure 1.**
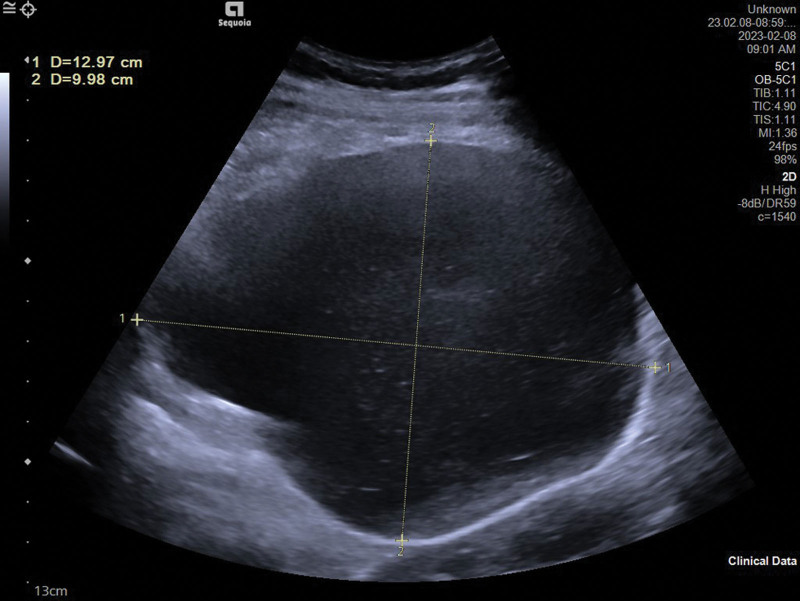
Left upper abdominal cyst of pregnant woman.

**Figure 2.**
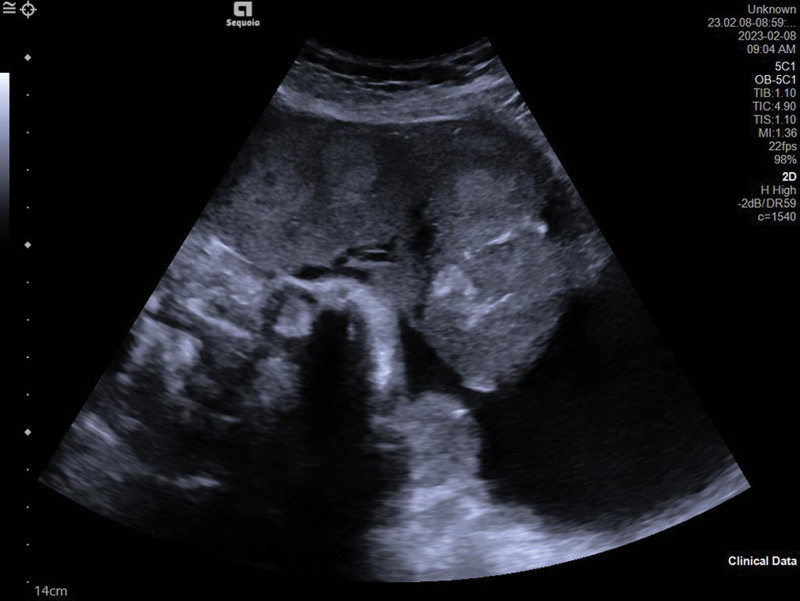
Uterine rupture: the rupture connecting cyst and amniotic cavity.

**Figure 3.**
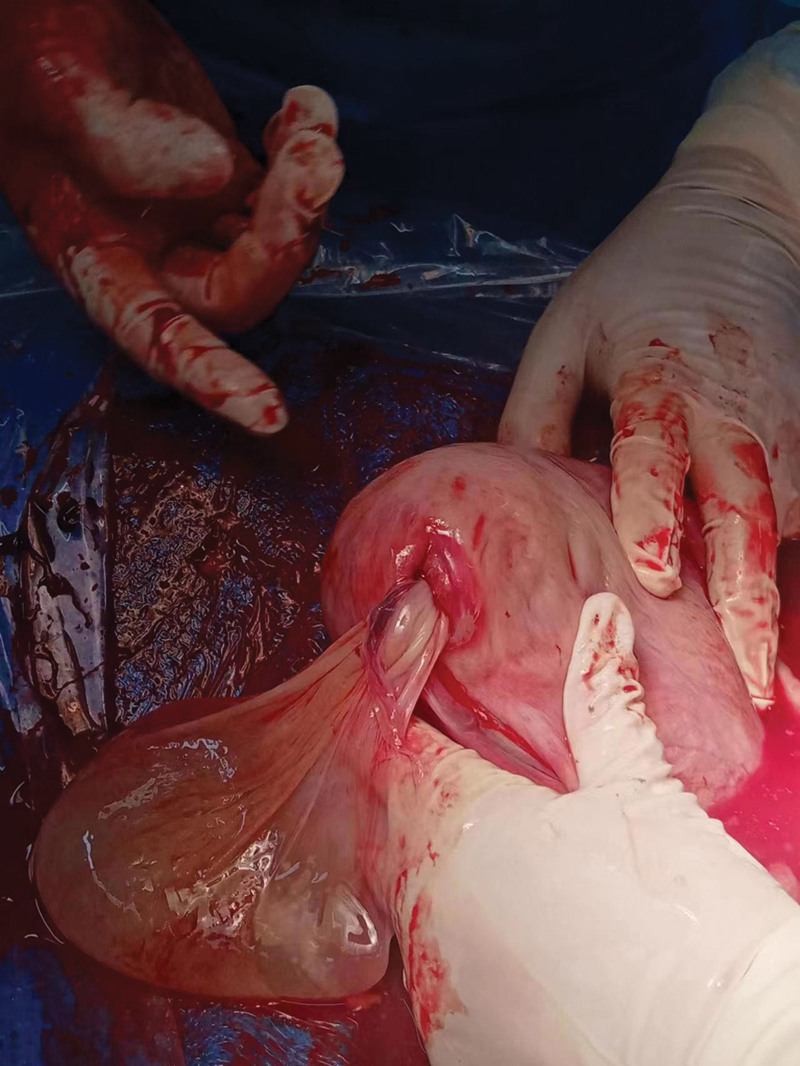
Intraoperative display: uterine rupture.

Informed consent was obtained from the patient for case publication. A 25-year-old patient was admitted to our hospital because she presented with slight abdominal pain in her 38th week of gestation. Ultrasound scan of the first doctor showed a single pregnancy, head position, cystic echo in the abdominal cavity (upper left of the uterus), the size of 99 × 78 × 56 mm, clear boundary, regular shape, poor sound transmission. Ultrasonic tip: abdominal cystic mass. The clinician reviewed the maternal history and found no evidence of a cystic mass in the abdominal cavity, so a second ultrasonography was requested by an expert sonographer, the results are as follows: there is a fetus in utero, and the fetal heart beats are visible. A cystic mass, approximately130 × 102 × 100 mm in size, was observed in the left upper abdomen of the pregnant woman and was attached to the amniotic cavity (Fig. 1). There was a continuous interruption of about 7 mm at the base of the left uterus (Fig. 2). No free effusion was found in the pelvic cavity. Ultrasonic tip: consider uterine rupture at the bottom of the left palace and amniotic sac. Finally, the patient underwent uterine repair and right salpingectomy. The surgery confirmed the diagnosis of uterine rupture (Fig. 3).

## 3. Discussion and conclusion

Spontaneous uterine rupture during pregnancy is a rare complication that carries a life-threatening risk for both mother and fetus. At present, the clear causes of uterine rupture include scar uterus, obstructive dystocia, improper use of uterine contraction drugs, obstetric surgical injury, abnormal uterine development, multiple uterine cavity operation history, etc,^[1–3]^ among which scar uterus is the most important cause.

This usually occurs when the uterine myometrium is weakened and thus is more prone to stress during labor. In an unscarred uterus, the incidence of uterine rupture is lower. The incidence of uterine rupture is not consistent in various countries, and the incidence of pregnancy-related uterine rupture is about 1.0/10,000 to 7.8/10,000.^[4]^ Uterine rupture risk factors of different countries, different periods each are not identical, the high-income countries uterine rupture during pregnancy is the most common in women with a history of cesarean section, and resource-poor countries is more common in obstructive dystocia, improper production division intervention or irregular operations, prenatal and lack of emergency obstetric treatment such as pieces of article.^[5]^

This case was managed at Jinan Maternity and Child Care Hospital Affiliated to Shandong First Medical University, a 25-year-old woman in her 38th week of gestation presented with slight abdominal pain of sudden onset 10 hours before. She had no history of cesarean section and uterine cavity operation.

The first ultrasound examination when ultrasound doctors saw the left abdominal cavity cystic mass when not considering the possibility of uterine rupture, also did not find the mass and the uterus is the real relationship. For the second time, experienced ultrasound experts were asked to examine the uterus and found that the uterus was ruptured. Finally, the operation confirmed that the uterus was completely ruptured (Fig. 3). A rupture of about 0.7 cm was found near the tubal orifice on the left side of the uterine fundus, with a few old blood stains, and the amniotic sac was prolapsed from the abdominal cavity about 9 × 8 × 8 cm in size. Uterine repair was performed. Postoperative examination showed that the left fallopian tube was unobstructed, and the residual horn uterus was seen on the right side. The base was wide and solid, and methylene blue injection and uterine cavity obstruction were performed. Studies have reported that abnormal fetal heart rate monitoring is the most common clinical manifestation of uterine rupture, with an incidence of 66% to 75%.^[6]^ When uterine rupture occurs, due to excessive contractions, excessive frequency, and blocked fetal blood supply, fetal heart rate changes or even fetal heart rate disappears. Electronic fetal heart rate monitoring graphics show severe variable deceleration and late deceleration. There was no abnormal manifestation of fetal heart rate monitoring in this patient. Savukyne et al^[7]^ reported that the most common symptom was abdominal pain, and the most sensitive indicator was abnormal fetal heart rate.

Literature shows that the early symptoms of noncesarean scar uterine rupture are often atypical, often with abdominal pain and abdominal distension are the main manifestations, which are easily confused with other gastrointestinal diseases.^[8]^ Because the initial abdominal pain sites of patients with different uterine rupture sites are also different, patients with uterine fundus or uterine horn rupture may first have upper abdominal pain, and patients with uterine posterior wall rupture may have lumbosacral pain, this leads to misdiagnosis and missed diagnosis.^[9,10]^ This patient does not have the typical symptoms and signs of uterine rupture, so the clinical diagnosis is difficult. At this time, ultrasonic diagnosis is particularly important, and its accuracy determines the clinical treatment. Timely and accurate second ultrasound examination saved the maternal and fetal life, and finally confirmed that the uterus was completely ruptured, presumably related to the congenital dysplasia of the uterus. The patient had no relevant data before the operation. Therefore, it is of great clinical significance for women of childbearing age to conduct a comprehensive prepregnancy examination before pregnancy to exclude the presence of congenital dysplasia of the uterus, and to be alert to uterine rupture during pregnancy. Through literature review and retrospective analysis, the reasons why the first ultrasound examination of this case was not clearly diagnosed were analyzed. First, The scan of cystic mass is not detailed and comprehensive enough, and the real relationship between the mass and the uterus is not found. Second, there was no comprehensive prepregnancy examination before pregnancy in this patient: uterine development did not suggest abnormalities Third, no stress test was normal, and mild abdominal pain did not attract enough attention from the ultrasound doctor. Lastly, the vigilance of uterine rupture is not high, lack of experience. Fortunately, both the patient and her baby survived the emergency intervention and all was well.

Therefore, regardless of whether it is combined with high-risk factors and whether the clinical symptoms are typical, we should always be alert to the possibility of uterine rupture, so as to achieve early identification and active treatment, so as to improve the prognosis of mother and child.

## Author contributions

**Conceptualization:** Linlin Wang.

**Data curation:** Yuxin Ma.

**Investigation:** Yin Yin.

**Project administration:** Juan Yin.

**Resources:** Zhihong Shi.

**Validation:** Zhihong Shi, Yuxin Ma.

**Visualization:** Linlin Wang.

**Writing—review & editing:** Yin Yin, Juan Yin.
